# A non-interventional cardiologist’s guide to coronary chronic total occlusions

**DOI:** 10.3389/fcvm.2024.1350549

**Published:** 2024-02-06

**Authors:** Lindsey Cilia, Michael Megaly, Rhian Davies, Behnam N. Tehrani, Wayne B. Batchelor, Alexander G. Truesdell

**Affiliations:** ^1^ Virginia Heart, Falls Church, VA, United States; ^2^ Inova Schar Heart and Vascular Institute, Falls Church, VA, United States; ^3^ Willis Knighton Medical Center, Shreveport, LA, United States; ^4^ Wellspan Health, York, PA, United States

**Keywords:** total coronary occlusion, coronary artery disease, chronic total occlusions, percutaneous coronary intervention, CABG, revascularization

## Abstract

Coronary chronic total occlusions (CTO) are present in up to one-third of patients with coronary artery disease (CAD). It is thus essential for all clinical cardiologists to possess a basic awareness and understanding of CTOs, including optimal evaluation and management. While percutaneous coronary intervention (PCI) for CTO lesions has many similarities to non-CTO PCI, there are important considerations pertaining to pre-procedural evaluation, interventional techniques, procedural complications, and post-procedure management and follow-up unique to patients undergoing this highly specialized intervention. Distinct from other existing topical reviews, the current manuscript focuses on key knowledge relevant to non-interventional cardiologists.

## Introduction

A chronic total occlusion (CTO) is defined as a 100% coronary artery occlusion that is non-acute and has been present for at least 3 months (1). Estimation of the occlusion duration is based upon first onset of classic anginal (or anginal equivalent) symptoms and/or history of myocardial infarction (MI) in the target vessel territory. Occluded coronary arteries discovered within 30 days of a MI are not considered CTOs, even though they may present technical revascularization challenges compared to acute lesions (2). There is an observed 15%–35% prevalence of CTOs in patients with CAD, increasing to 54%–89% for patients following coronary artery bypass grafting (CABG)—which has been noted to accelerate native vessel CAD and increase CTO prevalence post-operatively (3–8). The finding of a CTO during a diagnostic cardiac catheterization is also a common reason for referral to CABG, even though up to 30% of CTOs may still not be bypassed at surgery, as evidenced by data from the 2009 randomized SYNTAX (Synergy Between PCI with Taxus and Cardiac Surgery) trial (9, 10).

Fortunately, there have been significant advances in both equipment and procedural techniques for performing CTO PCI over the last decade. Yet, PCI referrals and attempt rates remain low—often influenced by past decades’ technological limitations. Whereas previous historical CTO PCI success rates hovered around 60%, success rates have improved to 80%–90% in contemporary CTO PCI registries (11–14). CTO PCI is most commonly performed to ameliorate anginal symptoms and improve quality of life, based on current data and expert recommendations (15). Anginal symptoms may be both “classic” and “non-classic” and include exertional chest (or jaw, neck, shoulder, arm, or abdominal) discomfort, or shortness of breath, and/or decreased exercise tolerance (16). Many patients with less-typical symptoms may incorrectly attribute these adverse feelings to non-cardiac disorders or to the “normal aging process”—as may their physicians. Patients may often understandably (and unfortunately) reduce their daily physical activity (to include simple activities of daily living) progressively over time to prevent or attenuate anginal burden, at the expense of quality of life (QOL) (17, 18).

## Indications: when to consider CTO PCI?

**Figure 1:**
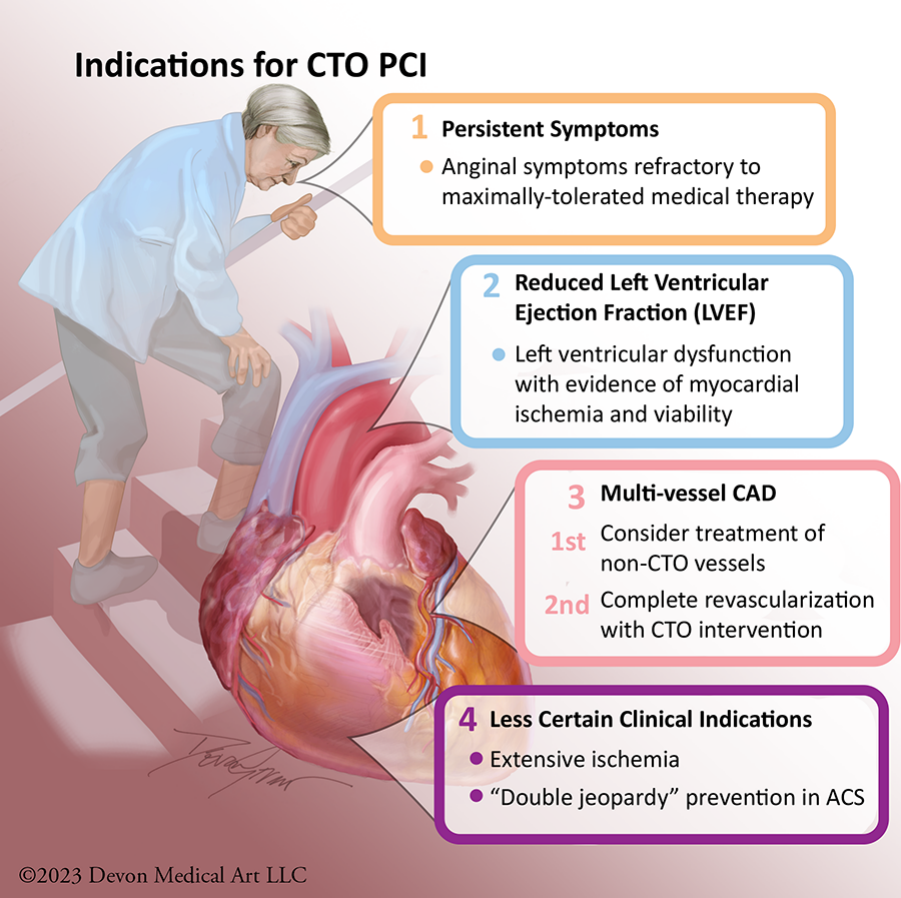
Indications for CTO PCI. Potential indications for CTO PCI primarily include: relief of angina, improvement in left ventricular systolic function, reduction of ischemia burden, prevention of “double jeopardy” in acute coronary syndrome, and complete revascularization.

Current expert consensus indications to consider recanalization of a CTO include: (1) to alleviate lifestyle-limiting symptoms and/or to increase exercise capacity; (2) to reduce the extent of ischemia as detected by non-invasive testing; (3) to improve dyspnea related to reduced left ventricular (LV) dysfunction with demonstrable evidence of viable myocardium; and (4) to improve long-term prognosis in patients with high-risk and prognostically-significant multi-vessel CAD (19–27). A less certain clinical indication is the prevention of a “double jeopardy” acute coronary syndrome event—occurring with acute occlusion of a non-CTO coronary artery providing collateral flow to a CTO myocardial territory, resulting in acute multivessel MI with risk for complete circulatory collapse due to cardiogenic shock (Figure 1) (28, 29).

The 2021 ACC/SCAI revascularization guidelines assign a Class IIb (treatment may be considered, but usefulness or efficacy is less well-established) recommendation for CTO PCI in patients with suitable anatomy and refractory angina despite medical therapy (30). The guidelines emphasize that the primary goal of CTO PCI should be to relieve symptoms, improve QOL, and increase exercise capacity (30). Based on the weight of existing randomized trial and observational data, anginal symptom improvement should remain the primary indication for consideration of CTO PCI.

## Clinical evidence for CTO PCI

Randomized clinical trials demonstrate that CTO PCI is most beneficial for symptom relief (31–34). The 2018 Euro-CTO (Randomized Multicentre Trial to Evaluate the Utilization of Revascularization or Optimal Medical Therapy for the Treatment of Chronic Total Coronary Occlusions) trial assessed health status difference at 12-months between optimal medical therapy (OMT) alone or OMT combined with PCI (11). CTO PCI was associated with significantly improved health status at follow-up compared to OMT alone. Patients with successful CTO PCI were noted to have fewer physical limitations, less angina, better mobility, and increased physical activity after revascularization as compared with patients treated with OMT alone. Additionally, observed periprocedural risks were low, and 12-month MACE rates were comparable to the OMT group.

The 2019 Decision-CTO (Drug-Eluting Stent Implantation Versus Optimal Medical Treatment in Patients With Chronic Total Occlusion) trial examined the outcomes of OMT alone compared to PCI coupled with OMT in patients with CTOs—and demonstrated low procedural complication rates and high procedural success but no difference in major adverse cardiovascular events (MACE) (35). Unfortunately, the study was limited by low power for clinical endpoints and was also terminated early due to slow enrollment and very high cross-over rates.

The 2016 EXPLORE (Evaluating Xience and left ventricular function in PCI on occlusiOns afteR STEMI) trial focused on LV function with concurrent CTO PCI for patients who presented with a ST-elevation MI and underwent primary PCI (36). While the trial had an overall low CTO PCI success rate of 73% and a high cross-over rate of 23%, a sub-study of patients with a left anterior descending coronary artery (LAD) CTO demonstrated benefit in LV ejection fraction (EF) improvement by cardiac MRI following successful CTO PCI—suggesting that CTO PCI to the LAD may improve not only clinical outcomes, but also LV geometry and function.

The 2017 OPEN-CTO (Outcomes, Patent Health Status, and Efficiency in Chronic Total Occlusion Hybrid Procedures) registry evaluated success rates, risks, and patient-reported benefits of contemporary CTO PCI (13). At one month following successful CTO PCI, significant improvements were seen in Seattle Angina Questionnaire (SAQ) QOL (49.4 ± 0.9–75.0 ± 0.7; *p* < 0.01), Rose Dyspnea Scale (2.0 ± 0.1–1.1 ± 0.1; *p* < 0.01), and Patient Health Questionnaire 8 (PHQ-8) (6.2 ± 0.2–3.5 ± 0.1; *p* < 0.01) parameters. Technical success rates in the registry were high, but complication rates were also higher than described for non-CTO PCI—highlighting the importance of careful evaluation of risks, benefits, and estimated technical success rates to most appropriately select optimal patients for CTO PCI and to best guide physician-patient shared decision-making conversations.

Overall, while there is abundant observational data suggesting that successful CTO PCI may be associated with improved clinical outcomes, prospective and randomized studies have been challenged by limitations in patient selection, trial design, and variable procedural success (37–40). Taken together, this data may be utilized to inform patient selection, education, pre-procedural counselling, and consent for CTO PCI—with evidence strongest at present for management of refractory anginal symptoms.

## Preparation for CTO PCI: detective work and medication optimization

Once the decision has been made to proceed to CTO PCI, an in-depth review of patient coronary anatomy is essential. This involves a thorough examination of all recent and historical invasive coronary angiography (which should be acquired at low magnification and without panning to facilitate optimal evaluation of collateral routes and with administration of intraocoronary nitroglycerine to improve distal vessel filling), non-invasive coronary computed tomography angiography (CCTA), and prior percutaneous or surgical intervention records (41). CCTA can provide critical information regarding the vessel course within the CTO segment—to include the degree and extent of calcification—and may be superior to coronary angiography for analysing proximal cap morphology (42). In some facilities, integration of CCTA with invasive coronary angiography may also be possible during PCI—thereby delineating the course of the occluded segment and potential crossing obstacles. Overall, a more complete understanding of patient coronary anatomy via careful review of both CCTA and invasive angiography significantly aids technical decision-making regarding CTO crossing strategies (and their hierarchy) and risk assessment, and thus procedural consent.

**Figure 2:**
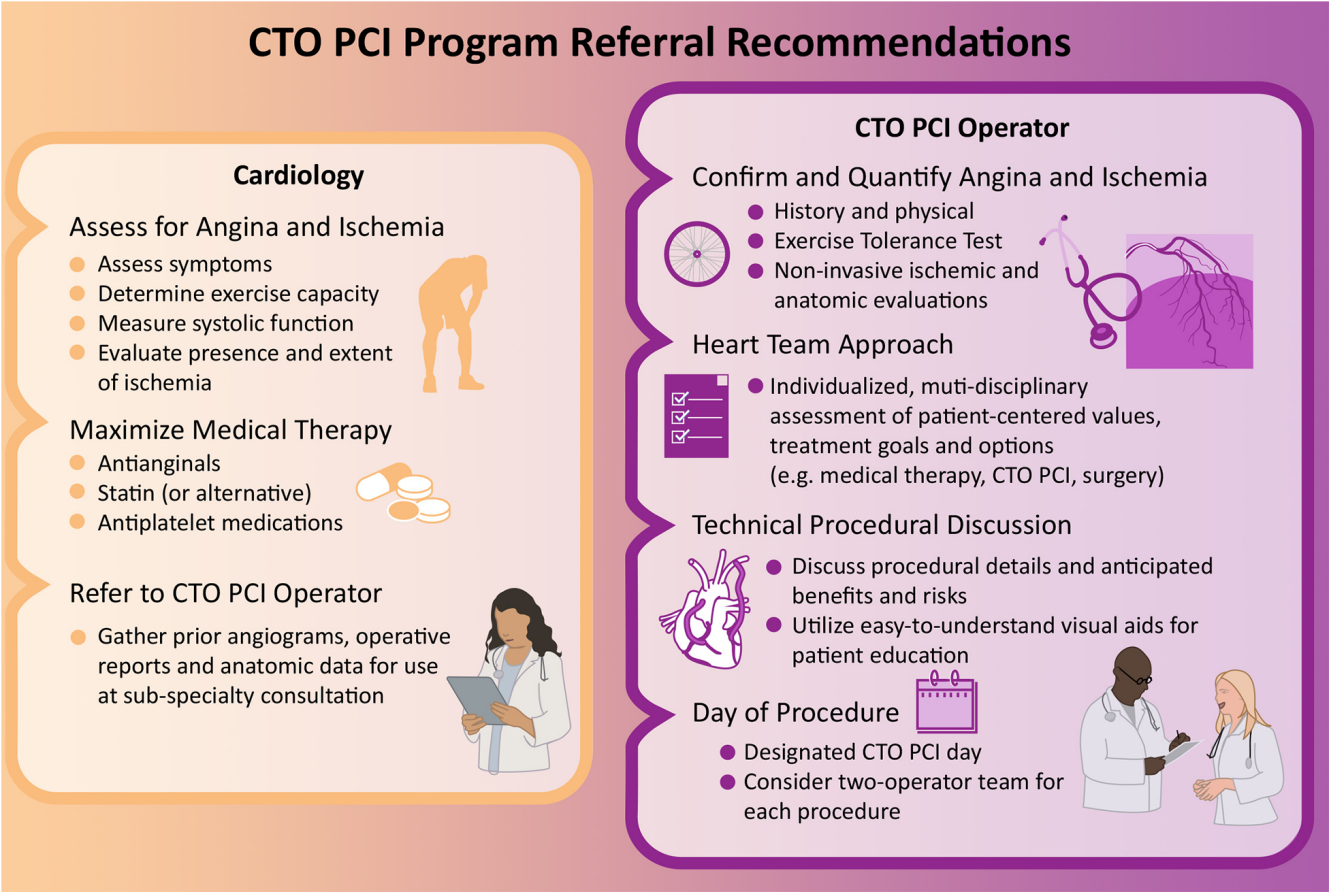
CTO PCI program referral recommendations. Evaluation and management of CTOs varies based on medical specialty and includes: assessment of angina and ischemia, review of patient comorbidities and values, maximization of medical therapy, referral to a CTO specialist, and consideration of PCI.

Since the principal current indication for CTO PCI is symptom relief, we have adopted an algorithmic approach to optimal anti-anginal medication initiation in the outpatient setting prior to consideration of CTO PCI. These anti-anginal medications include a beta-blocker such as Metoprolol Succinate, a long-acting nitrate such as Imdur, a calcium channel blocker such as Amlodipine, and the metabolic modulator Ranolazine—all aimed at optimizing myocardial oxygen supply/demand. Only after patients are up-titrated over time to maximally tolerated doses of these medications (or prove intolerant to doses which adequately control symptoms due to adverse side effects), do we proceed with CTO PCI. Physicians who care for patients with CTOs should strongly consider referral to a CTO PCI specialist for further evaluation unless they are asymptomatic and with good exercise tolerance, normal EF, and minimal ischemic myocardium. When possible, it is advised that clinicians caring for patients undergoing evaluation for potential CTO PCI obtain prior invasive and non-invasive imaging studies in advance of CTO PCI specialty consultation and attempt medical optimization. Ultimately it is incumbent upon CTO PCI teams to complete any of this unfinished diagnostic or therapeutic work prior to pursuing invasive intervention (Figure 2).

## CTO crossing strategies and scoring systems

Given the complex technical strategies needed to perform CTO PCI safely and effectively and the enhanced procedural risks associated with these interventions, it is advised that patients undergoing evaluation for these procedures be referred to specialized and experienced interventional cardiologists at high-volume and high-complexity medical centers. Successful CTO PCI often requires multiple radial and/or femoral arterial access points to aid in vessel visualization and lesion crossing. There are essentially four techniques to traverse a CTO—antegrade wire escalation (AWE), antegrade dissection and re-entry (ADR), retrograde wire escalation (RWE), and retrograde dissection re-entry (RDR) (12, 15). In other words, CTO operators can cross CTO lesions four possible ways—from the antegrade direction straight through the blockage (“intraplaque” wire tracking within the occlusive intima-based plaque), from the antegrade direction “around” the blockage (“extraplaque” wire tracking outside the plaque but still contained within the adventitial layer), or from the retrograde direction via collateral vessels either straight through the blockage or “around” the blockage (31). Each individual crossing strategy and additional access point adds to procedural complexity and risk—advocating for both appropriate clinical indications and appropriate operator training and experience.

Multiple different CTO crossing expert consensus algorithms currently exist (e.g., Hybrid, Asia-Pacific, EuroCTO) and most recently a Global algorithm merging the best of each of these primary regional protocols has been proposed (43). Despite technical differences prioritizing one (initial) crossing strategy over another, all of the individual protocol share key guiding principles—to include a focus on the complementary nature of antegrade and retrograde wiring and reentry strategies, the importance of efficient switching between alternative crossing techniques to optimize success and shorten procedure time and radiation dose, and the critical importance of intracoronary imaging (15). Intracoronary imaging is vital for the optimal performance of all PCI, and CTO PCI in particular—for evaluation of intraplaque vs. extraplaque tracking, preintervention lesion assessment (to include assessment of plaque composition and follow-on optimal plaque modification technique), lesion preparation and stent deployment and optimization, and assessment of postprocedure endpoints and complications (44).

Various scoring systems have been developed to predict the technical success of CTO PCI and guide risk/benefit analysis and doctor/patient decision-making. Each scoring system considers multiple variables—to include both demographic and angiographic features. Two of the most common scoring systems to predict technical success are the J-CTO (Multicenter Chronic Total Occlusion Registry in Japan) and PROGRESS-CTO (Prospective Global Registry for the Study of Chronic Total Occlusion Intervention) scores (45, 46).

The J-CTO score predicts the likelihood of crossing the CTO lesion within 30 min. It includes five factors (each worth one point when present): occlusion length ≥20 mm, blunt stump appearance of the proximal cap of the occlusion, calcification within the lesion segment, presence of a >45-degree bend within the CTO, and prior failed PCI attempt. A J-CTO score of 0 is considered “easy”, 1 is intermediate, 2 is difficult, and ≥3 is very difficult, with the probability of a technically successful procedure described as 97.8%, 92.3%, 88.4%, and 73.3%, respectively (45). The PROGRESS-CTO score uses four independent variables to predict the likelihood of successful CTO recanalization: ambiguous proximal cap of the CTO, moderate or severe vessel tortuosity, circumflex artery as the target CTO vessel, and lack of “interventional collaterals” to support a retrograde procedural technique—with similarly graded success rates (which are also improving over time) as the J-CTO score (46, 47).

## Complications: prevention, recognition, and management

CTO interventions are among the most complex and high-risk PCI procedures performed in the modern cardiac catheterization lab (48). Complications, while uncommon, can be catastrophic if not successfully anticipated, prevented, recognized, and managed (49). As presented in this document, meticulous procedural planning—to include detailed assessment of appropriate clinical indications, anticipated clinical benefit, anatomic complexity, and patient-specific procedural risk—is a critical aspect of CTO PCI (50). A detailed description of the technical aspects of management of specific CTO PCI complications has been well described elsewhere (51).

Many experienced CTO centers presently achieve high success rates (85%–90%) with low (2%–3%) risks of major periprocedural complications (52). By comparison, technical success rates >95% and complication rates <2% have been reported for non-complex non-CTO PCI—compared to success rates <60% (and >6% incidence of emergent CABG) at the dawn of PCI in the 1980s (53, 54). Still, despite lower technical success rates, equivalent MACE rates have been reported between the currently more routinely encountered complex (as opposed to non-complex) non-CTO PCI and CTO PCI (4.1% vs. 5.0% in a large recent single-center registry) (55, 56). Ultimately while there have been iterative advances in CTO PCI equipment and techniques and improvements in success and complication rates (in parallel with extension of procedures to increasingly more complex patient and lesion subsets), the benefit-to-risk ratio remains less favorable compared with non-CTO PCI and may be best limited to patients with refractory angina and dyspnea or high ischemic burden, and performed by high-volume, high-experience, CTO PCI teams and institutions (57).

The decision to pursue CTO PCI—as with all medical interventions—depends on the balance of estimated risk and anticipated benefit. When indicated and successful, CTO PCI may offer relief of angina, improvement in QOL, and possible improvements in myocardial function, exercise capacity, prevention of arrhythmias, and long-term survival (58–60). In-hospital procedural complications are similar to non-CTO PCI and include death, MI, stroke, perforation, pericardial tamponade, side branch occlusion, coronary dissection, major bleeding and need for blood transfusion, contrast-induced nephropathy, vascular surgery repair, and urgent CABG (51). In the initial 2017 report of the multi-center OPEN-CTO Registry, in-hospital mortality occurred in 0.9% of patients, myocardial infarction in 2.6%, emergency CABG in 0.7%, and coronary perforation requiring treatment in 4.8% (13). Scoring systems such as the PROGRESS-CTO complication risk score can facilitate estimation of these periprocedural risks of death, MI, urgent target vessel revascularization, tamponade requiring intervention, and stroke in patients undergoing CTO PCI (overall 2.1% in PROGRESS-CTO) and thus inform physician-patient pre-procedure counseling and shared decision-making (61).

## Is CTO PCI appropriate for every operator and every center?

While CTO PCI may not be appropriate for every operator or every institution, therapy awareness and the option to undergo this intervention should be available to all eligible patients as part of a multidisciplinary Heart Team management model (30, 62–64). Compared with complex non-CTO PCI, this intervention involves very unique techniques and equipment—to include specialized lesion crossing strategies, specialty wires and microcatheters, and re-entry devices (48). Dedicated CTO PCI operators need to be facile with all four of the main lesion crossing strategies (as well as additional sub-strategies) unique to CTO PCI (48, 65). Finally, in order to achieve high success rates, operators should possess the skillset to rapidly and fluidly transition between multiple alternative AWE, ADR, RWE, and RDR strategies—which often only occurs with dedicated individual and team training and accumulated experience. Additional optimal CTO PCI program requirements have been well described elsewhere (15, 41).

Karacsonyi et al. recently analyzed the association between operator volume and procedural outcomes of 7,035 CTO PCI procedures performed between 2012 and 2021 at 30 centers and observed that higher-volume operators (>60 CTO PCI cases/year) performed higher complexity procedures with higher rates of technical and procedural success (87.9%) (66). In another analysis by Zein et al. of 7,389 CTO PCIs performed between 2010 and 2018 at 46 sites in Michigan, combined operator and hospital CTO PCI experience was directly related to procedural success but not to major adverse cardiac events—although notably only 4 institutions performed >50 CTO PCIs per year (with 81% procedural success among this higher-volume center cohort) (67).

Presently more than two-thirds of practicing US interventional cardiologists perform fewer than 100 total (non-CTO) PCI procedures annually and nearly 50% perform fewer than 50 total (non-CTO) PCI procedures annually—with unsurprisingly lower mortality noted for high-volume vs. low-volume operators (1.53% vs. 1.86%) (68, 69). Additionally, while a volume-outcome relationship has been noted for a variety of complex PCI lesion subsets in multiple studies, non-CTO PCI volume, expertise, and outcomes do not directly translate to CTO PCI safety and success (70–72). Together, this data may advocate for the regionalization of CTO PCI care to teams of experienced high-volume CTO operators at experienced high-volume CTO centers (Figure 2).

## Knowledge gaps and research opportunities

Multiple questions remain regarding the potential benefits of CTO PCI beyond angina relief and increase in exercise capacity. Ongoing randomized controlled trials (RCT), such as the NOBLE-CTO (Nordic-Baltic Randomized Registry Study for Evaluation of PCI in Chronic Total Coronary Occlusion; NCT03392415) and ISCHEMIA-CTO (Nordic and Spanish Randomized Trial on the Effect of Revascularization or Optimal Medical Therapy of Chronic Total Coronary Occlusions With Myocardial Ischemia; NCT03563417) trials, expected to complete enrollment in 2027 and 2028, respectively, may help address these unanswered questions and further inform future myocardial revascularization guidelines.

## Conclusions

CTOs are common in daily practice and new and evolving treatment options now exist for lesions that were once considered “untreatable”. While many patients are asymptomatic with medical therapy alone, others suffer lifestyle-limiting angina which may significantly curtail their activities of daily living. Although there are multiple potential advantages to CTO intervention, the most proven current indication is for symptom relief and quality of life improvement rather than survival benefit. Due to higher procedural complexity and risk and a need for greater operator technical expertise, CTO PCI may be most appropriately performed by specially-trained and experienced teams at high-volume cardiac catheterization laboratories.
